# Vagal baroreflex activation resulting in acute coronary stent thrombus associated with myocardial infarction: a case report

**DOI:** 10.1186/1471-2261-14-131

**Published:** 2014-09-27

**Authors:** Yan-Yan Jing, De-Cai Luan, Liu-Dong Li

**Affiliations:** Department of Cardiology, Yuhuangding Hospital, Qingdao Medical College, Qingdao University, Yantai, Shandong Province China

**Keywords:** Vagal baroreflex activation, Acute coronary stent thrombosis, Myocardial infarction

## Abstract

**Background:**

Percutaneous coronary intervention with stenting in patients with coronary atheromatous stenosis carry an inherent risk of affecting the baroreflex-mediated regulation of hemodynamic alterations, especially for heart rate and blood pressure. To the best of our knowledge, the vagal baroreflex activation associated with acute coronary stent thrombosis in patients who have undergone percutaneous coronary intervention has not been previously reported.

**Case presentation:**

In the present article, we report a case of a Chinese patient (a 75-year-old male) with coronary artery disease who presented with hemodynamic alterations as a complication of vagal baroreflex activation after implantation of overlapping stents, followed by stent thrombosis associated with myocardial infarction.

**Conclusion:**

The patient’s vagal baroreflex sensitivity increased after the coronary stenting procedure. He was successfully treated with intra-aortic balloon pump therapy. Because of its rarity, this case is being reported to emphasize the importance of using intra-aortic balloon pump therapy.

## Background

Percutaneous coronary intervention (PCI) has become an important procedure for the treatment of myocardial infarction. Although autonomic activity, baroreflex sensitivity, and hemodynamic alterations resulting from vagal baroreflex activation (VBA) have been reported in patients who have undergone carotid angioplasty and stenting [1–3], there are few data available about the hemodynamic alterations resulting from VBA in PCI patients [4]. The present article reports the case of a patient who developed an acute stent thrombosis associated with myocardial infarction, which resulted from VBA after PCI. To the best of our knowledge, the autonomic activity, baroreflex sensitivity, and hemodynamic alterations resulting from VBA in patients treated with PCI have not been previously reported.

## Case presentation

A 75-year-old Chinese man who had been experiencing chest pain for 10 years presented at our institution. He was moderately active, doing odd jobs around the house, and accelerating angina over the past 2 years. The patient had been taking aspirin, nifedipine for high blood pressure and metformin for diabetes mellitus type 2 for almost 10 years. He smoked one pack of cigarettes per day for about 40 years before quitting the previous year. His past history was remarkable for chronic obstructive pulmonary disease.

At the time of admission, the physical examination showed heart rate (HR), 67 beats/min (bpm); blood pressure (BP), 156/89 mmHg; and blood glucose, 6.85 mmol/L. An electrocardiogram (ECG) showed a sinus rhythm with ST depression and T-wave inversion when chest pain occurred. A complete blood count and a basal metabolic profile were examined; the evaluation of kidney function, blood acid, base balance, and blood sugar levels showed that there were all within a normal range. The treadmill exercise test was positive, and showed ST depression on the ECG when chest pain or shortness of breath occurred. The patient’s response to clopidogrel therapy was determined by testing for CYP450 genetic polymorphism, which showed non-resistance to such therapy. Testing for CYP450 genetic polymorphism was to predict response to clopidogrel [5].

The patient was prepared for coronary angiography (and stent placement if necessary). Before the procedure, the patient’s spontaneous baroreflex sensitivity (BRS), heart rate variability (HRV), and blood pressure variability (BPV) were measured (Table 1). Six hours before the patient underwent coronary angiography, he was given 300 mg aspirin and 300 mg clopidogrel. An initial dose of heparin (100U/kg) was intravenously given before the procedure; an additional dose of heparin at 500U would be given if the procedure lasted 1 hour. The activated clotting time was maintained at 325 s.

**Figure 1:**
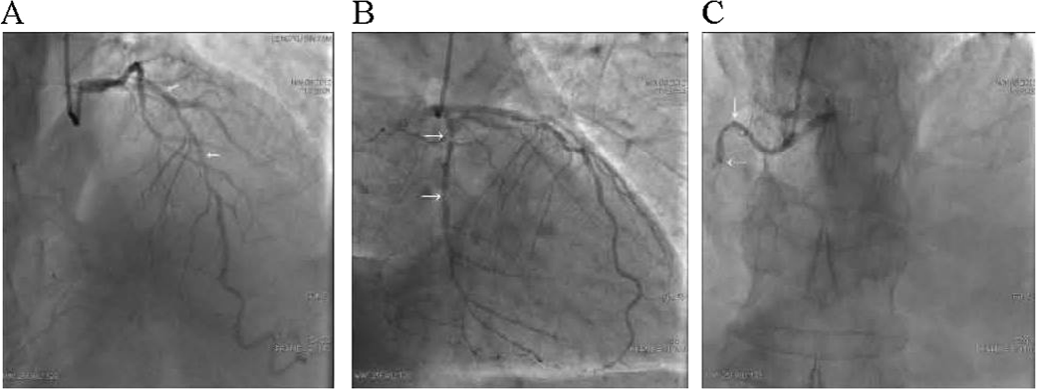
**Coronary angiogram showing diffuse stenosis in the middle segments of the left anterior descending artery (A, arrows), the left circumflex artery (B, arrows) and with occlusion in the right coronary artery (C, arrows).**

The coronary angiogram was done through the radial approach. The angiogramrevealed a proximal 90% diffuse stenosis with calcification in the middle segment of the left anterior descending artery (Figure 1A), and 95% stenosis with calcification in the left circumflex artery (Figure 1B). The right coronary artery was observed to have >80% diffuse stenosis with heavy calcification and occlusion in the middle segment (Figure 1C).

**Figure 2:**
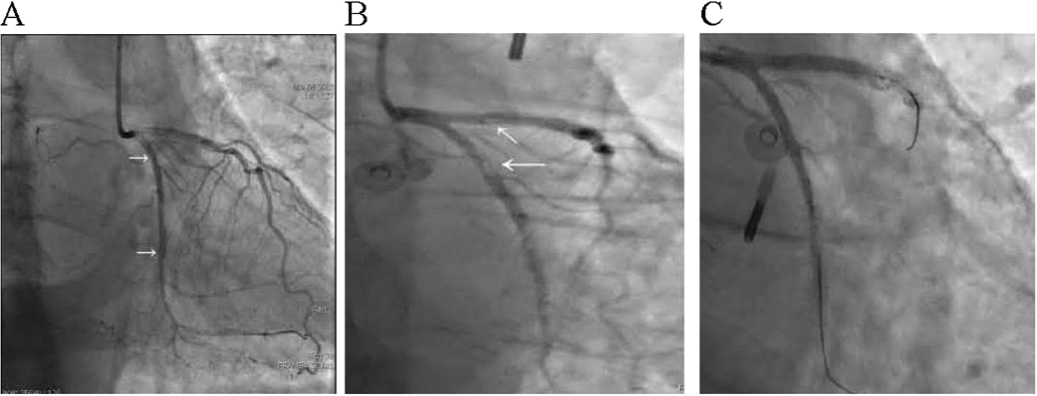
**Coronary angiogram showing no stenosis following overlapping stent implantation (A, arrows), thrombus in the stents in the anterior descending and circumflex arteries (B, arrows) and thrombus disappearing following intra-aortic balloon pump therapy (C).**

Two overlapping sirolimus-eluting stents (Cypher; Cordis, Miami, USA.), mounted on a balloon catheter, were inserted without a gap into the left anterior descending artery (stents were 36 mm in length and 3 mm in diameter) and the left circumflex artery (stents 14 mm in length and 3 mm in diameter), respectively (Figure 2A). The stents were advanced into the narrowed section of the arteries. When the stents were positioned, the balloon was inflated. To ensure full expansion of the stents, the stent balloon was gradually inflated to 14 atm in 10 to 40 seconds. Because of chronic total occlusion in the right coronary artery, we attempted to pass a wire through the lesion but failed. Considering that collateral circulation was already present, we abandoned the stent placement into the right coronary artery. After the stenting procedure, the thrombolysis in myocardial infarction (TIMI) grade was 3, and no cineangiographic characteristics of thrombus were present. The procedure was uneventful without a special condition for the patient.

**Table 1:** **Heart rate, systolic/diastolic blood pressure, heart rate variability, blood pressure variability and spontaneous baroreflex sensitivity before and after coronary artery stenting**

	Pre-stenting	Post-stenting
HR (beats/min)	67	39
SBP (mmHg)	156	87
DBP (mmHg)	89	52
HRV (ms^2^)	1019	1728
BPV (mmHg^2^)	10.1	16.8
BRS (ms/mmHg)	7.2	12.6

BPV: Blood pressure variability; BRS: Spontaneous baroreflex sensitivity; DBP: Diastolic blood pressure; HR: Heart rate; HRV: Heart rate variability; SBP: Systolic blood pressure.

Approximately 20 minutes after the procedure (18:40), the patient developed nausea, vomiting, sweating and slow breathing associated with diaphoresis and pale complexion. His blood pressure was 87/52 mmHg and heart rate was 39 bpm. At that time, the patient was conscious and had no chest pain. No evidence of bleeding or hematoma was found. The ECG monitor showed no change in ST/T waves. The HRV and BRS were, however, found to be greatly elevated (Table 1) compared with the levels measured before stenting, suggesting VBA. After administration of 1.5 L intravenous saline with dopamine and atropine, the BP and HR slightly improved.

However, at 22:50, the patient complained of precordial chest pain and diaphoresis, and presented drowsy, cold and clammy extremities. His peripheral pulses were not palpable, and blood pressure was not recordable. The ECG monitors showed ST elevation in leads V1, V2, V3 and V4; reciprocal ST depression in leads I, aVL and aVF; and sinus bradycardia with first-degree heart block. The cardiac troponin I (cTnI) testing value was 0.06 ng/mL (normal value 0.04 ng/mL). A diagnosis of myocardial infarction associated with first-degree heart block and cardiogenic shock was made.

Immediately a repeat coronary angiogram was done through the femoral approach, and the left anterior descending and circumflex arteries, into which the stents had been placed, were reexamined. The angiogram revealed thrombotic occlusion of the planted stents (Figure 2B) in both the left anterior descending and circumflex arteries, with TIMI flow grades of 1 and 0, respectively. The thrombus burden was graded as G3 in the circumflex artery and as G4 in the left anterior descending artery. No evidence of artery dissection was found.

An intra-aortic balloon pump (IABP) was immediately inserted. The TIMI flow grade showed no improvement after runs of aspiration in the two arteries; at that time the patient was still in cardiogenic shock. An intracoronary balloon was placed at the location where the thrombi adhered in the left anterior descending and circumflex arteries. While continuing counterpulsation of the IABP, the thrombi in the stents disappeared shortly afterward (Figure 2C). The patient was given tirofiban, aspirin, clopidogrel and heparin; the balloon counterpulsation was maintained until the patient’s heart rate normalized and blood pressure stabilized. The patient was discharged with a normal echocardiographic evaluation, and was asymptomatic at follow-up.

## Discussion

The hemodynamic alterations involving arterial baroreflex and cardiac autonomic control in patients undergoing with carotid angioplasty or stenting are extensively reported [1–3]; conversely, there are few reports in patients who undergo PCI with angioplasty and stenting for coronary stenosis. In this study, we report that VBA caused hemodynamic alterations after a PCI procedure, followed by stent thrombosis associated with myocardial infarction. The patient was successfully treated with intra-aortic balloon counterpulsation. To the best of our knowledge, this is the first case describing VBA associated with acute coronary stent thrombosis after PCI.

In the clinical experience, patients developing VBA with bradycardia and hypotension during PCI procedure usually are instantaneous or last for a little while. Interestingly, our patient developed such hemodynamic alterations 20 minutes after the procedure. This extremely uncommon incident raised two questions: 1) whether the vagal stimulation resulted in the subsequent myocardial infarction or the myocardial infarction resulted in hemodynamic alterations; and 2) whether it might just be vice versa and the observed hemodynamic changes could be the result of a stent thrombosis.

The patient initially presented nausea, vomiting and slow breathing associated with diaphoresis and pale complexion, followed by bradycardia and hypotension. These symptoms typically result from vagal stimulation. Although occlusion might also activate cardiac vagal afferents, at that moment the patient had neither chest pain nor discomfort and showed no change in ECG. In addition to these clinical features, which excluded the possibility of myocardial infarction, measurements of BRS, HRV and BPV can be used to evaluate changes in baroreflex control and cardiac autonomic activity [1]. The results showed that the patient’s BRS level increased, but his HR and BP levels decreased, suggesting the occurrence of VBA. The baroreflex is a very important regulatory mechanism for BP and HR [3]. Therefore, the initial hemodynamic alterations resulted from the stenting procedure and caused a subsequent stent thrombosis associated with myocardial infarction.

The stenting-caused hemodynamic alterations may be due to the effects of direct artery baroreceptor stimulation. Coronary stenting in patients with coronary atheromatous stenosis offers an opportunity to elucidate the cardiovascular autonomic response to direct intravascular stimulation of the baroreceptors. VBA development in patients undergoing coronary stenting could be due to distension of the coronary artery, which leads to stimulation of the baroreceptors, an increase in cardiac parasympathetic stimulation and a decrease in adrenergic output to the peripheral vasculature. The continuous force to the vessel wall from the implanted stents increases vagal activity and baroreflex sensitivity. Stress and pain may also stimulate VBA, which affects hemodynamic stability and leads to nausea, vomiting, diaphoresis, paleness, decreasing blood pressure and/or drowsiness with cold and clammy extremities.

Our patient presented a series of clinical features of VBA resulting from the stenting procedure. The alterations in HR, BP, and breathing components were initially due to VBA because, at that moment, the patient showed no evidence of myocardial infarction, except for BRS and HR/BP levels in opposite manner. With continuing vagal activity and baroreflex sensitivity, the development of hemodynamic alterations, including hypotension and decreased blood flow in the coronary artery, was subsequently complicated by thrombosis in the implanted stents, as confirmed by the repeat angiogram. As a result of stent thrombosis or artery occlusion, myocardial infarction developed as confirmed by ECG and cTnI testing.

In consideration for VBA, it is necessary to exclude the possibility of cardiac tamponade, especially in a patient who has undergone PCI with stenting. The examination of BRS and HRV is a method of identifying patients at risk for cardiovascular disease associated with autonomic dysfunction [1]. In our patient, the opposite manner of BRS and HR/BP levels supported that the hemodynamic alterations resulted from VBA. Management of the VBA includes the expansion of blood volume and the administration of atropine, dopamine, and painkillers. These treatments generally relieve the patient’s symptoms within a short period. However, our patient had a series of hemodynamic alterations, followed by thrombosis within the implanted stents. The thrombi in the stents resulted in myocardial infarction with cardiogenic shock, which was managed with IABP therapy.

IABP is an important therapy for increasing myocardial oxygen supply and decreasing myocardial oxygen demand [6], although its use is at the very least controversial. In our patient, IABP was used due to the failure of thrombus aspirations.

The overlapping stents might have contributed to the risk of thrombosis within the stents, resulting in myocardial infarction. Stenosis of the coronary artery is a main cause of myocardial infarction [7], because the smallest minimal luminal diameter is located within the zone of the stent overlap [8]. Therefore, the initial hemodynamic alterations caused by VBA were greatly associated with the subsequent stent thrombosis in our patient.

The mechanism of stent thrombosis is multifactorial factors [9]. Severe vagal stimulation is an important factor, which acts to lower heart rate, decrease blood pressure and slow blood flow, resulting in stent thrombosis or ST-elevation myocardial infarction. Myocardial reinfarction caused by stent thrombosis is associated with a larger thrombus burden [10, 11], and stent thrombosis after primary PCI may be associated with a particularly poor prognosis [9]. Dual antiplatelet therapy with aspirin and clopidogrel is the standard care for most patients undergoing PCI [9]. Regarding clopidogrel therapy, more attention should be given to the conversion of CYP2C19 enzyme into its active metabolite [9, 5].

## Conclusion

Coronary stenting caused an increase in baroreflex sensitivity and vagal activation in our patient. The patient’s condition was complicated by stent thrombosis followed by myocardial infarction. The patient was successfully treated with intra-aortic balloon pump therapy. Because of its rarity, this case is being reported to emphasize the importance of using intra-aortic balloon pump therapy in patients such as ours.

## Consent

Written informed consent was obtained from the patient for publication of this case report and any accompanying images. A copy of the written consent is available for review by the Editor-in-Chief of this journal.

## Authors’ information

Yan-Yan Jing and De-Cai Luan share the first author.

## Abbreviations

BPV: Blood pressure variability
BRS: Spontaneous baroreflex sensitivity
HRV: Heart rate variability
IABP: Intra-aortic balloon pump
PCI: Percutaneous coronary intervention
VBA: Vagal baroreflex activation.
